# Clinical Phenotype of DiGeorge Syndrome with Negative Genetic Tests: A Case of DiGeorge-Like Syndrome?

**DOI:** 10.1155/2015/938074

**Published:** 2015-12-17

**Authors:** Gianluigi Laccetta, Benedetta Toschi, Antonella Fogli, Veronica Bertini, Angelo Valetto, Rita Consolini

**Affiliations:** ^1^Pediatric Department, Faculty of Medicine, Rheumatology and Clinical Immunology Unit, Pisa University, 56126 Pisa, Italy; ^2^Medical Genetics Unit, Children Department, Pisa University, 56126 Pisa, Italy; ^3^Cytogenetics and Molecular Genetics Unit, Children Department, Pisa University, 56126 Pisa, Italy

## Abstract

We report a case of DiGeorge-like syndrome in which immunodeficiency coexisting with juvenile idiopathic arthritis, congenital heart disease, delay in emergence of language and in motor milestones, feeding and growing problems, enamel hypoplasia, mild skeletal anomalies, and facial dysmorphisms are associated with no abnormalities found on genetic tests.

## 1. Introduction

DiGeorge syndrome (DGS) is usually caused by 22q11.2 deletion; the most common deletion includes loss of* TBX1* gene which is an important transcription factor for the development of the heart, thymus, parathyroid glands, palate, and teeth: thus, haploinsufficiency of* TBX1* is thought to be the greatest cause of the disorder [1, 2]. Rarely, most of DGS phenotypes are well explained by* TBX1* gene mutations [3].

We describe a patient with clinical findings of DGS and negative molecular genetic tests.

## 2. Case Report

The patient was a 5-year-old male who suffered from pain on his right knee since one year prior to presentation. His right knee was swollen and flexed, his right lower limb was hypotrophic, and his left leg was 1 centimeter shorter in length than his right one. The child had valgus heels and turned his feet inward during the walk; he also had difficulties in walking and climbing stairs. ANA (anti-nuclear antibodies), ACA (anti-centromere antibodies), RF (rheumatoid factor), and ASO (antistreptolysin O) titers were negative and CRP (C-reactive protein) was 1.86 mg/dL (normal value: less than 0.50 mg/dL). X-rays of the knee were normal; ultrasonography and magnetic resonance imaging exhibited a distended anterior joint recess filled with fluid. Juvenile idiopathic arthritis was diagnosed (Juvenile Arthritis Damage Index, JADI = 3) and the patient required oral ibuprofen treatment (30 mg/kg per day divided into 2 doses), intra-articular steroids (20 mg of triamcinolone hexacetonide in 0.5 mL of lidocaine 1%), and serial arthrocentesis. There were neither ocular manifestations of iridocyclitis nor uveitis; the child only had hyperopia requiring prescription lenses.

The prenatal course of the child was characterized by the diagnosis of mild hypoplastic left heart, hypoplastic aortic arch, and persistent left superior vena cava draining into the coronary sinus. When the child was 13 days old he successfully underwent nonemergency repair of his hypoplastic aortic arch using autologous pericardium and surgical obliteration of patent ductus arteriosus with extracorporeal circulation. The patient was also diagnosed with bicuspid aortic valve, dysplastic mitral valve, left-ventricular false tendon, and tricuspid insufficiency; he also had a perimembranous ventricular septal defect which spontaneously closed. The child was diagnosed with progressive aortic recoarctation of periductal type when he was 5 months old and underwent cardiac catheterization and balloon angioplasty using properly sized balloons (CB-Balt 4 × 20 mm, 6 × 25 mm, and 8 × 20 mm); balloon angioplasty reduced peak-to-peak gradient from 47 to 0 mmHg. The patient was treated with captopril (0.3 mg/kg, 3 times a day) up to when he was 5 years old and his parents were told to give the child antibiotic prophylaxis for bacterial endocarditis in case of need. On physical examination performed at our institution, a 2/6 holosystolic murmur was heard over heart; cardiac frequency (108 bpm), blood pressure (100/65 mmHg), oxygen saturation (SaO_2_ 98%), and respiratory rate (21 times/min) were normal. Electrocardiogram showed sinus rhythm, incomplete right bundle branch block, and abnormal ventricular repolarization.

The early course of the child was also characterized by feeding and growing difficulties as he was below the third percentile in weight during his first three years of life; thus, the child was diagnosed with ankyloglossia and underwent surgical treatment twice when he was 3 years old. Rhinoscopy and oropharyngoscopy performed at our institution were normal and physical examination showed that the patient was between the third and the fifteenth percentile both in height (103 cm) and in weight (15.8 kg) according to 2007 WHO growth charts; thus, feeding and growing difficulties were probably caused by ankyloglossia.

The patient also had delay in emergence of language: he was able to speak at 3 years 2 months and needed speech therapy. The child reported neither sensorineural nor conductive hearing loss as otoscopy, tympanogram, and audiometric evaluation were normal. The patient also had delay in motor milestones as he could sit at 12 months and was able to walk at 2 years; when first evaluated by neuropsychiatrists, the child was aged 4 and exhibited shyness, difficulty with social interactions, and deficits in fine motor coordination. Neuropsychiatrists noted difficulties in the area of verbal communication, reading decoding, grammatical skills, and spelling; cognitive assessments were performed using the Wechsler Preschool and Primary Scale of Intelligence-Third Edition (WPPSI-III): full scale IQ was moderately below average (score: 78), performance IQ was just below average (score: 88), and verbal IQ was significantly below average (score: 69). Results of cognitive tests made us suspect that delays in emergence of language and in motor milestones were associated; delay in emergence of language was partially due to ankyloglossia.

With regard to the immune function, serial lymphocyte counts showed that the patient had impaired T-cell production not improving over time; the restricted repertoire of T cells caused dysregulation in B cell compartment; in fact total B cells were reduced. Immunologic evaluation at our institution at 5 years of age revealed a low number of white blood cells; percentage of lymphocytes and that of absolute lymphocyte count were below the normal range. Percentage and number of CD3+, CD4+, and CD8+ cells were decreased for age but CD4+ to CD8+ ratio was normal; CD19+ cells were also reduced. Immunologic screening showed a high level of CD16+/56+ cells, just like patients with DGS [4]. Laboratory findings also demonstrate an accelerated conversion of naïve T cells to memory T cells, as typical of patients with DGS [5]. The patient had a low percentage of *γ*-globulin in serum protein electrophoresis but total serum proteins were in the normal range; IgA, IgM, and IgG levels were decreased for age. Results of immunologic laboratory tests were the ones in Table 1.

The child suffered from recurrent sinusitis, otitis media, and lower respiratory infections in his early years of life because of impaired lymphocytes production, hypogammaglobulinemia, and feeding difficulty.

The child had enamel hypoplasia and caries experience on his course but calcium-phosphorus metabolism was normal.

The child also had clinodactyly of the bilateral little fingers and protuberant ears; no abnormalities were detected by abdominal ultrasonography performed at our institution. Laboratory tests showed normal hepatic and renal functions.

With regard to congenital heart disease, immunodeficiency coexisting with juvenile idiopathic arthritis, delay in emergence of language and in motor milestones, mild skeletal anomalies (bilateral clinodactyly of the fifth fingers), facial dysmorphism (protuberant ears), and enamel hypoplasia, DGS was considered. Ankyloglossia has not been reported in DGS but it should be considered a kind of oral median dysplasia, just like cleft palate; bicuspid aortic valve has been described in a small percentage of patients with DGS [1]. Therefore, FISH (Fluorescent In Situ Hybridization) analysis with Vysis N25 (22q11.2)/ARSA probe was performed but no microdeletions were identified; automatic sequencing of the* TBX1* gene coding sequence made by PCR and 3130xl Genetic Analyzer identified no mutations and computer molecular dynamics simulations showed neither reduced nor modified function of the corresponding T-box transcription factor* Tbx1*. Finally, Array-comparative genomic hybridization (Array-CGH) analysis, performed by standard procedures using both 8X60K and 4X180K oligo platforms (Agilent Technologies, Santa Clara, CA, USA), showed neither microdeletions nor microduplications.

## 3. Discussion

This infant's presentation is consistent with DGS but genetic tests do not confirm this diagnosis; thus, our patient should be considered a case of DiGeorge-like syndrome. Five disorders (Smith-Lemli-Opitz syndrome, Alagille syndrome, VATER association, Goldenhar syndrome, and CHARGE syndrome) have overlapping features with DGS but our patient has been diagnosed with none of them [1]. At present, the child is followed up by healthcare providers from many specialties.

## Figures and Tables

**Table 1:** Immunologic profile of the patient.

Panel	Result	Normal range
CD3+ cells (cells/*μ*L)	950	1092–1216
CD3+ cells (%)	53.9	62.0–69.0
CD4+ cells (cells/*μ*L)	467	529–705
CD4+ cells (%)	26.5	30.0–40.0
CD8+ cells (cells/*μ*L)	351	440–564
CD8+ cells (%)	19.9	25.0–32.0
CD16+/56+ cells (cells/*μ*L)	490	141–264
CD16+/56+ cells (%)	27.8	8.0–15.0
CD19+ cells (cells/*μ*L)	315	370–493
CD19+ cells (%)	17.9	21.0–28.0
CD4+ to CD8+ ratio	1.33	1.30–1.50
Naïve (CD45RA+CD62L+) T CD4+ cells (%)	19.4	24.3–81.0
Naïve (CD45RA+CD62L+) T CD8+ cells (%)	18.3	19.9–66.4
Memory (CD45RA−CD62L+) T CD4+ cells (%)	37.4	3.5–36.2
Memory (CD45RA−CD62L+) T CD8+ cells (%)	59.3	1.9–34.2
IgA (mg/dL)	35	109 ± 35
IgM (mg/dL)	48	85 ± 26
IgG (mg/dL)	586	975 ± 248
